# Musculoskeletal System and Connective Tissue Related Hospital Admission in England and Wales Between 1999 and 2019: An Ecologic Study

**DOI:** 10.7759/cureus.32453

**Published:** 2022-12-12

**Authors:** Saja Mustafa Ali, Abdallah Y Naser, Aseel Ghazi Alghanemi, Amal Khaleel AbuAlhommos, Marwa Sabha, Moaath K Mustafa Ali, Sara Ibrahim Hemmo, Ahmad M Alrajeh, Jaber S Alqahtani, Abdulelah M Aldhahir, Hassan Abu Rokbah

**Affiliations:** 1 Department of Internal Medicine, Saint Agnes Hospital, Baltimore, USA; 2 Department of Applied Pharmaceutical Sciences and Clinical Pharmacy, Faculty of Pharmacy, Isra University, Amman, JOR; 3 Department of Family Medicine, King Abdulaziz University Faculty of Medicine, Jeddah, SAU; 4 Department of Pharmacy Practice, King Faisal University, Al-Ahsa, SAU; 5 Division of Rheumatology and Clinical Immunology, University Hospitals (UH) Cleveland Medical Center, Case Western Reserve University School of Medicine, Ohio, USA; 6 Department of Hematology and Medical Oncology, Taussig Cancer Institute, Cleveland Clinic, Ohio, USA; 7 Department of Respiratory Care, College of Applied Medical Sciences, King Faisal University, Al-Ahsa, SAU; 8 Department of Respiratory Care, Prince Sultan Military College of Health Sciences, Dammam, SAU; 9 Department of Respiratory Therapy, Faculty of Applied Medical Sciences, Jazan, SAU; 10 Department of General Surgery, Al Noor Specialist Hospital, Makkah, SAU

**Keywords:** admissions, trends, musculoskeletal diseases, hospitalization, connective tissue diseases

## Abstract

Background

There is a lack of data describing inpatient hospitalization trends for musculoskeletal and connective tissue diseases in the United Kingdom.

Aim

We aim to provide a comprehensive analysis of the trends of musculoskeletal and connective tissue disease related hospitalizations between 1999 and 2019 in England and Wales.

Method

We conducted an ecologic study. The data were obtained from the Hospital Episode Statistics database in England and the Patient Episode Database in Wales between 1999 and 2019. We used ICD-10 (International Classification of Diseases, 10th Revision) codes M00-M99 to identify hospital admissions.

Results

The total annual hospital admission rate increased from 1,303.63 (95% CI: 1,300.55-1,306.71) in 1999 to 2,479.09 (95% CI: 2,475.14-2,483.04) in 2019 per 100,000 persons (p<0.01). The ICD-10 categories other joint disorders, osteoarthritis, and other dorsopathies accounted for 19.6%, 19.6%, and 18.6% of hospitalizations, respectively. Advanced age groups experienced a larger increase in hospitalization rates (128.6% in the age group of 75 years and above vs. 45.9% in the age group below 15 years). Females contributed to 57.7% of hospitalizations and experienced a larger increase in hospitalization rate compared to males (103.8% vs. 73.8%).

Conclusion

Between 1999 and 2009, the hospitalization rate for musculoskeletal and connective tissue diseases has steadily increased in England and Wales. However, the rate has plateaued or declined in many of musculoskeletal and connective tissue diseases between 2010 and 2019. Due to the chronicity of these diseases, their significant morbidity, and significant long-term disability, national interventions are needed to mitigate the effects of the increased cost of treatment.

## Introduction

Pharmacologic and non-pharmacologic interventions have revolutionized the treatment of many musculoskeletal system and connective tissue related diseases over the last two decades [1]. Diagnosing and treating these diseases are challenging and commonly require comprehensive approaches involving patients, physicians, and ancillary staff. Multi-disciplinary teams delivering pharmacotherapies, including glucocorticoids, disease-modifying agents, and biological agents, are the mainstay treatment in many connective tissue diseases. Non-pharmacological interventions are essential in treating musculoskeletal-connective tissue diseases, which include surgery and physical and occupational therapy [2]. The complexity of musculoskeletal and connective tissue diseases arise from multiple factors, including the frequent atypical presentation of the diseases, the significant disease burden, the associated comorbidities, and the treatment-related adverse events [3]. The development of targeted and biological therapies used in immune-mediated inflammatory diseases has significantly improved outcomes [4], yet these patients remain at risk of therapy-related side effects and financial toxicity. Patients suffering from musculoskeletal and connective tissue diseases may have prolonged survival and complications, requiring long-term emotional and physical support. The burden of such diseases accumulates with age, causing significant social and physical impact and commonly resulting in work disability [5].

In the United Kingdom (UK), musculoskeletal and connective tissue diseases are one of the leading causes of national health service expenditure, with it being the fourth leading cause of budget expenditure [6]. It was estimated to affect 10 million adults and around 12,000 children in England nowadays [6]. However, to our knowledge, there are no comprehensive studies investigating hospitalization trends for musculoskeletal and connective tissue disease in the UK. In this study, we conducted an ecologic study to investigate the trends of musculoskeletal and connective tissue disease related hospitalizations between 1999 and 2019 in England and Wales.

## Materials and methods

As previously described in recent epidmeiological studies that were conducted in the UK using publicly available hospital admissions data [7-20], we conducted this ecologic study using publicly available data extracted from the Hospital Episode Statistics (HES) database in England [21] and the Patient Episode Database in Wales (PEDW) for the period between April 1999 and April 2019 [22]. The HES and PEDW databases contain data on hospital admissions for patients with musculoskeletal system and connective tissue diseases from all age groups. Admissions were categorized based on gender, and age was presented as four groups: below 15 years, 15-59 years, 60-74 years, and 75 years and above. The International Classification of Diseases (ICD) system diagnostic codes for musculoskeletal system and connective tissue diseases (M00-M99) were used to identify hospital admission of interest in the databases (M00-M02: Infectious arthropathies; M04-M04: Autoinflammatory syndromes; M05-M14: Inflammatory polyarthropathies; M15-M19: Osteoarthritis; M20-M25: Other joint disorders "Acquired deformities of fingers and toes, Other acquired deformities of limbs, Disorder of patella, Internal derangement of knee, and other specific joint derangements"; M26-M27: Dentofacial anomalies [including malocclusion] and other disorders of jaw; M30-M36: Systemic connective tissue disorders; M40-M43: Deforming dorsopathies; M45-M49: Spondylopathies; M50-M54: Other dorsopathies "Cervical disc disorders, Thoracic, thoracolumbar, and lumbosacral intervertebral disc disorders, Other and unspecified dorsopathies, not elsewhere classified, and Dorsalgia"; M60-M63: Disorders of muscles; M65-M67: Disorders of synovium and tendon; M70-M79: Other soft tissue disorders "Soft tissue disorders related to use, overuse and pressure, Other bursopathies, Fibroblastic disorders, Shoulder lesions, Enthesopathies, lower limb, excluding foot, and Other enthesopathies"; M80-M85: Disorders of bone density and structure; M86-M90: Other osteopathies "Osteomyelitis, Osteonecrosis, Osteitis deformans [Paget's disease of bone], and Other disorders of bone"; M91-M94: Chondropathies; M95-M95: Other disorders of the musculoskeletal system and connective tissue; M96-M96: Intraoperative and postprocedural complications and disorders of musculoskeletal system, not elsewhere classified; M97-M97: Periprosthetic fracture around internal prosthetic joint; M99-M99: Biomechanical lesions, not elsewhere classified). The HES and PEDW data are routinely checked to ensure validity and accuracy [21,23]. To calculate the annual hospitalization rate for these disorders, we obtained midyear population estimates between 1999 and 2019 from the Office for National Statistics (ONS) [24]. The Scientific Research Ethics Committee at the Faculty of Pharmacy in Isra University, Amman, Jordan, reviewed the study protocol, and the study was considered exempt because of the de-identified nature of the data.

Statistical analysis

Hospital admission rates with 95% confidence intervals (CIs) were calculated using the number of musculoskeletal system and connective tissue related admissions divided by the midyear population size. We used Poisson models to assess the trend in hospitalization due to the nature of the data (count data). We conducted three Poisson regression models with the admission rate as the dependent variable, and the years of admission for the total population, gender (across the years), and age group (across the years) as the independent variables. The number of hospital admissions linked to each disease category for each age group among males was divided by the midyear population for males of the same age group in the same year to calculate annual hospital admissions rates. Same procedure was used to calculate the admission rates stratified by gender. A similar method was used for females to calculate annual hospital admissions rates for each disease category. The chi-square test was used to determine the difference in hospital admission rates. We analyzed data using SPSS version 25 (IBM Corp, Armonk, NY, USA) [25].

## Results

The total annual hospital admission number for diseases of the musculoskeletal system and connective tissue increased by 116.8%, from 679,716 in 1999 to 1,473,566 in 2019, representing a 90.2% increase in hospital admission rate (from 1,303.63 [95% CI: 1,300.55-1,306.71] in 1999 to 2,479.09 [95% CI: 2,475.14-2,483.04] in 2019 per 100,000 persons, trend test, p<0.01). Most diseases of the musculoskeletal system and connective tissue had a steeper increase in the rate of hospitalization until 2008/2009, after which they began to stabilize, and for some disorders (inflammatory polyarthropathies, other joint disorders, deforming dorsopathies, spondylopathies, other dorsopathies, and disorders of synovium and tendon), a decline in the admission rate was observed starting from the year 2015/2016 (Table 1). The most common causes of hospital admissions were the following ICD-10 categories: other joint disorders (19.6%), osteoarthritis (19.6%), other dorsopathies (18.6%), and other soft tissue disorders (15%) (Table 1).

**Table 1:** Percentage of the musculoskeletal system and connective tissue diseases’ hospital admissions from the total number of admissions per ICD-10 code in England and Wales between 1999 and 2019. ICD-10, International Classification of Diseases, 10th Revision

ICD-10 code	Description	Percentage of total number of admissions
M00-M02	Infectious arthropathies	0.6%
M05-M14	Inflammatory polyarthropathies	7.9%
M15-M19	Osteoarthritis	19.6%
M20-M25	Other joint disorders (Acquired deformities of fingers and toes, other acquired deformities of limbs, disorder of patella, internal derangement of knee, and other specific joint, derangements)	19.6%
M30-M36	Systemic connective tissue disorders	2.0%
M40-M43	Deforming dorsopathies	1.0%
M45-M49	Spondylopathies	5.0%
M50-M54	Other dorsopathies (Cervical disc disorders, thoracic, thoracolumbar, and lumbosacral intervertebral disc disorders, other and unspecified dorsopathies, not elsewhere classified, and dorsalgia)	18.6%
M60-M63	Disorders of muscles	0.7%
M65-M67	Disorders of synovium and tendon	3.2%
M70-M79	Other soft tissue disorders (Soft tissue disorders related to use, overuse and pressure, other bursopathies, fibroblastic disorders, shoulder lesions, enthesopathies, lower limb, excluding foot, and other enthesopathies)	15.0%
M80-M85	Disorders of bone density and structure	3.4%
M86-M90	Other osteopathies (Osteomyelitis, osteonecrosis, osteitis deformans [Paget's disease of bone], and other disorders of bone)	2.0%
M91-M94	Chondropathies	0.7%
M95-M95	Other disorders of the musculoskeletal system and connective tissue	0.2%
M96-M96	Intraoperative and postprocedural complications and disorders of musculoskeletal system, not elsewhere classified	0.4%
M99-M99	Biomechanical lesions, not elsewhere classified	<0.1%

**Figure 1:**
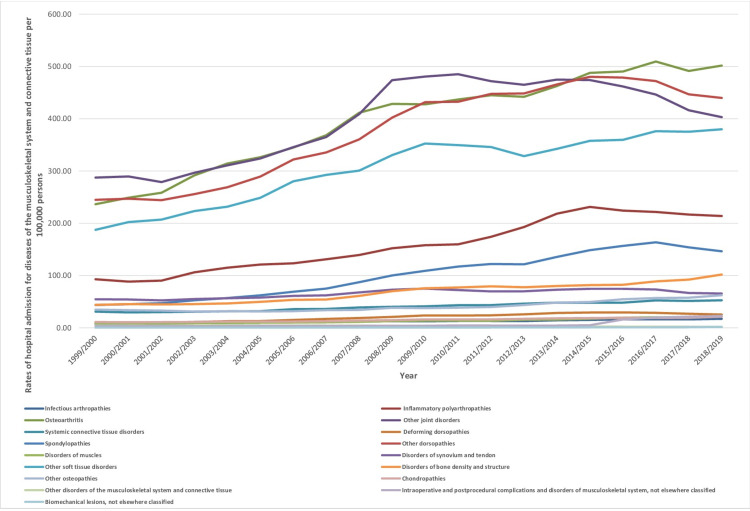
Hospital admission rates for musculoskeletal system and connective tissue diseases in England and Wales stratified by type between 1999 and 2019.

Over the past two decades, the hospital admission rate for all musculoskeletal system and connective tissue diseases categories increased, except for the ICD-10 category “Other disorders of the musculoskeletal system and connective tissue,” which decreased (Figure 1). The highest increase in the hospital admissions rate was noted in the following ICD-10 categories: biomechanical lesions, not elsewhere classified, intraoperative and postprocedural complications and disorders of the musculoskeletal system, not elsewhere classified, deforming dorsopathies, disorders of muscles, disorders of bone density and structure, inflammatory polyarthropathies, infectious arthropathies, osteoarthritis, other soft tissue disorders with 6.27-fold, 5.79-fold, 1.45-fold, 1.44-fold, 1.32-fold, 1.31-fold, 1.15-fold, 1.12-fold, and 1.03-fold increase in the admission rate, respectively (Table 2). Using quadrant trend analysis, we found that the change was noticed for infection arthropathies in the year 2012/2013 and for other dorsopathies in the year 2014/2015.

**Table 2:** Percentage change in the hospital admission rates for musculoskeletal system and connective tissue diseases in England and Wales between 1999 and 2019.

Disorder	Rate of diseases in 1999 per 100,000 persons (95% CI)	Rate of diseases in 2019 per 100,000 persons (95% CI)	Percentage change from 1999 to 2019	Percentage change from 1999 to 2009	Percentage change from 2009 to 2015	Percentage change from 2015 to 2019	P-value†
Infectious arthropathies	8.08 (7.84–8.33)	17.34 (17.01–17.68)	114.5%	58.2%	19.6%	7.1%	≤0.001
Inflammatory polyarthropathies	92.80 (91.98–93.63)	213.99 (212.82–215.17)	130.6%	64.0%	46.4%	-4.6%	≤0.001
Osteoarthritis	236.47 (235.15–237.79)	501.64 (499.84–503.43)	112.1%	81.2%	14.0%	2.3%	≤0.001
Other joint disorders (acquired deformities of fingers and toes, other acquired deformities of limbs, disorder of patella, internal derangement of knee, and other specific joint derangements)	287.37 (285.92–288.82)	403.03 (401.42–404.64)	40.2%	64.9%	-1.4%	-12.7%	≤0.001
Systemic connective tissue disorders	31.09 (30.61–31.57)	52.81 (52.23–53.40)	69.9%	29.3%	16.6%	9.4%	≤0.001
Deforming dorsopathies	10.39 (10.11–10.67)	25.42 (25.02–25.83)	144.7%	101.9%	23.6%	-13.7%	≤0.001
Spondylopathies	43.64 (43.07–44.21)	146.34 (145.37–147.31)	235.3%	129.7%	36.3%	-6.6%	≤0.001
Other dorsopathies (cervical disc disorders, thoracic, thoracolumbar, and lumbosacral intervertebral disc disorders, other and unspecified dorsopathies, not elsewhere classified, and dorsalgia)	244.93 (243.59–246.27)	439.75 (438.07–441.44)	79.5%	64.2%	11.3%	-8.1%	≤0.001
Disorders of muscles	8.81 (8.56–9.07)	21.53 (21.16–21.91)	144.3%	45.9%	25.3%	10.4%	≤0.001
Disorders of synovium and tendon	54.63 (54.00–55.27)	65.60 (64.95–66.25)	20.1%	33.4%	-0.5%	-12.1%	≤0.001
Other soft tissue disorders (soft tissue disorders related to use, overuse and pressure, other bursopathies, fibroblastic disorders, shoulder lesions, enthesopathies, lower limb, excluding foot, and other enthesopathies)	187.45 (186.27–188.62)	379.78 (378.22–381.35)	102.6%	76.3%	1.5%	5.6%	≤0.001
Disorders of bone density and structure	43.85 (43.28–44.42)	101.83 (101.02–102.64)	132.2%	59.8%	7.9%	23.6%	≤0.001
Other osteopathies (osteomyelitis, osteonecrosis, osteitis deformans [Paget's disease of bone], and other disorders of bone)	34.78 (34.28–35.29)	62.36 (61.72–62.99)	79.3%	10.3%	31.4%	14.1%	≤0.001
Chondropathies	11.26 (10.97–11.54)	21.34 (20.97–21.71)	89.6%	31.2%	16.1%	10.7%	≤0.001
Other disorders of the musculoskeletal system and connective tissue: (other acquired deformities of musculoskeletal system and connective tissue)	4.50 (4.32–4.69)	1.95 (1.84–2.06)	-56.7%	-11.5%	-35.1%	-9.3%	≤0.001
Intraoperative and postprocedural complications and disorders of musculoskeletal system, not elsewhere classified	3.36 (3.20–3.52)	22.83 (22.45–23.21)	579.4%	19.5%	26.5%	43.7%	≤0.001
Biomechanical lesions, not elsewhere classified	0.21 (0.17–0.25)	1.53 (1.43–1.63)	627.3%	218.3%	53.1%	64.4%	≤0.001

**Figure 2:**
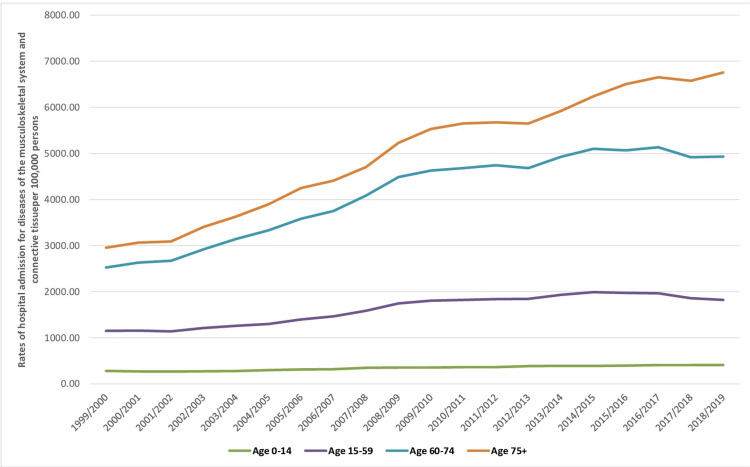
Hospital admission rates for musculoskeletal system and connective tissue diseases in England and Wales stratified by age group between 1999 and 2019.

Hospital admissions related to musculoskeletal system and connective tissue diseases varied among different age groups, with almost half of admission occurring in the age group of 15-59 years (47.6%). This was followed by the age group of 60-74 years (29.6%), the age group of 75 years and above (19.8%), and, lastly, the age group below 15 years (3.0%). The rate of hospital admissions for musculoskeletal system and connective tissue diseases in patients aged below 15 years increased by 45.9% (from 281.05 [95% CI: 277.76-284.35] in 1999 to 410.19 [95% CI: 406.37-414.02] in 2019 per 100,000 persons, trend test, p<0.001). The rate of hospital admissions in patients aged 15-59 years increased by 58.4% (from 1,149.31 [95% CI: 1,145.58-1,153.03] in 1999 to 1,820.48 [95% CI: 1,816.01-1,824.94] in 2019 per 100,000 persons, trend test, p<0.001). The rate of hospital admissions in patients aged 60-74 years increased by 95.3% (from 2524.77 [95% CI: 2513.09-2536.45] in 1999 to 4931.36 [95% CI: 4917.40-4945.32] in 2019 per 100,000 persons, trend test, p<0.001). Lastly, the rate of hospital admissions in patients aged 75 years and above increased by 128.6% (from 2,954.05 [95% CI: 2,937.27-2,970.82] in 1999 to 6,753.64 [95% CI: 6,731.81-6,775.46] in 2019 per 100,000 persons, trend test, p<0.001) (Figure 2).

**Figure 3:**
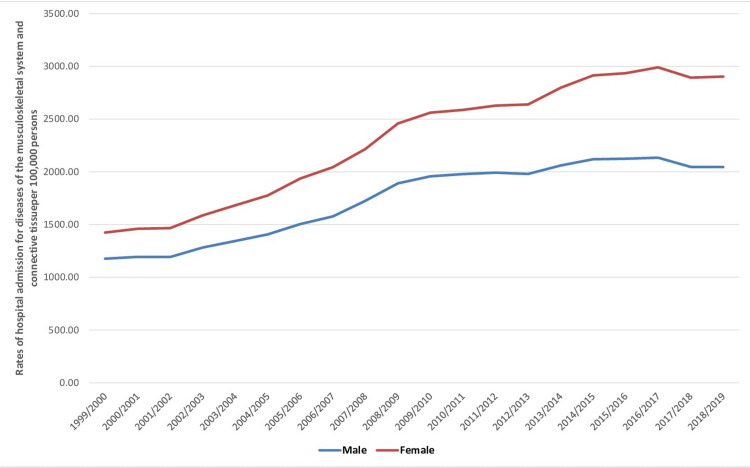
Hospital admission rates for musculoskeletal system and connective tissue diseases in England and Wales stratified by gender between 1999 and 2019.

A total of 22,612,354 hospital admissions related to musculoskeletal system and connective tissue diseases occurred in England and Wales throughout the study duration. Females contributed to 13,056,148 hospital admissions (57.7%), averaging 652,807 admission per year. Musculoskeletal system and connective tissue diseases hospital admission rate in females increased by 103.8% (from 1,424.55 [95% CI: 1,420.06-1,429.05] in 1999 to 2,902.55 [95% CI: 2,896.54-2,908.55] in 2019 per 100,000 persons, trend test, p<0.001). On the other hand, the hospital admission rate in males increased by 73.8% (from 1,176.68 [95% CI: 1,172.49-1,180.87] in 1999 to 2,045.13 [95% CI: 2,040.01-2,050.25] in 2019 per 100,000 persons, trend test, p<0.001) (Figure 3).

Diseases of the musculoskeletal system and connective tissue admission rate by gender

Females experienced a larger increase in hospital admission rates in the following musculoskeletal system and connective tissue hospital ICD-10 categories: inflammatory polyarthropathies, osteoarthritis, other joint disorders, systemic connective tissue disorders, deforming dorsopathies, spondylopathies, other dorsopathies, disorders of synovium and tendon, other soft tissue disorders, disorders of bone density and structure, chondropathies, and intraoperative and postprocedural complications and disorders of the musculoskeletal system, not elsewhere classified. On the other hand, males had a larger increase in hospital admission rates in the following ICD-10 categories: infectious arthropathies, disorders of muscles, other osteopathies, other disorders of the musculoskeletal system and connective tissue, and biomechanical lesions, not elsewhere classified were higher.

Diseases of the musculoskeletal system and connective tissue admission rate by age group

Most musculoskeletal system and connective tissue hospital admissions increased with advancing age groups. That includes the following ICD-10 categories: osteoarthritis, other joint disorders, systemic connective tissue disorders, spondylopathies, other dorsopathies, disorders of muscles, other soft tissue disorders, disorders of bone density and structure, other osteopathies, intraoperative and postprocedural complications and disorders of musculoskeletal system, not elsewhere classified, and biomechanical lesions, not elsewhere classified. Furthermore, hospital admissions due to infectious arthropathies, and deforming dorsopathies were more common among the age groups 75 years and above and 60-74 years, compared to age groups below 15 years and 15-59 years. In contrast, hospital admissions due to inflammatory polyarthropathies, and disorders of synovium and tendon were higher in the following descending sequence of age groups: 60-74 years, 75 years and above, 15-59 years, and below 15 years. In addition, hospital admissions due to other musculoskeletal system and connective tissue diseases were higher in the following descending sequence of age groups: 15-59 years, 60-74 years, below 15 years, and 75 years and above. On the other hand, hospital admissions due to chondropathies were more common among those below 15 years.

## Discussion

Musculoskeletal and connective tissue diseases encompass various conditions that commonly run chronic courses and are associated with significant morbidity and mortality, resulting in increased risk of hospital admissions. Our study found a significant overall increase in hospital admissions rates related to musculoskeletal system and connective tissue diseases in England and Wales between 1999 and 2019. However, most diseases of the musculoskeletal system and connective tissue had a steeper increase until 2008/2009, after which they began to stabilize, and for some disorders, a decline in the admission rate was observed in the last five years of the study.

Our study found that around half of musculoskeletal system and connective tissue disease hospital admissions occurred in the age groups 60-75 years and 75 years and above. Moreover, these age groups have experienced the highest increase in admission rates compared to younger age groups. This can be attributed partly to the changes in the UK's age demographics. In the UK, the life expectancy of males and females has improved from 74.7 and 79.4 in the 1997-1999 period to 79.4 and 83.1 in the 2017-2019 period, respectively [26]. The current estimated number of people aged 65 years and above is nearly 12 million in the UK In 2030, one-fifth of the population is predicted to be above 65 years [27]. This improvement in life expectancy resulted in an increased prevalence of chronic diseases, including musculoskeletal and connective tissue disorders [28]. By 2035, more than half of the elderly in the UK are predicted to have multi-comorbidity, defined as the coexistence of two or more chronic medical diseases, and arthritis' prevalence is projected to increase by 92% compared to 2015 [28]. This group of patients is at high risk of hospital admissions and prolonged hospital stays [28]. For example, in 2018, falling among the elderly was the most common cause of emergency hospital admissions in the UK [29]. The impact of musculoskeletal system and connective tissue diseases on the elderly could be devastating as it may lead to loss of mobility with subsequent physical and psychological disability [27]. Moreover, the elderly frequently experience treatment delays and interruptions, resulting in recurrent hospitalization and increased mortality [30].

Previous research that used publicly available data revealed that hospital admissions have been rising across the board for various organ diseases [7,8,10,12,30]. The cause of this pervasive increase is unknown. In previous and current studies, we observed that older age groups' admissions had increased the most, possibly due to the "aging" of the UK population, as described above. Moreover, the increase in outpatient healthcare services utilization is an emerging challenge in the UK [31]. The number of patients waiting for consultant-led elective care increased from 3.25 million in September 2015 to 6 million in January 2022. In January 2022, patients are waiting for 13 weeks on average to receive treatment in the outpatient setting [31]. This striking increase in healthcare utilization might result in a shift in care from elective to emergent, as patients waiting for outpatient treatment might experience complications or worsening of their symptoms, leading to hospitalization. Another potential cause of increased hospitalization across organs is the increased availability and complexity of treatments and associated side effects secondary to advancements in these treatments.

Our analysis showed that females accounted for more than half of musculoskeletal and connective tissue disease admissions, with an almost 100% increase in admission rate between 1999 and 2019. The highest increase occurred in inflammatory polyarthropathies (130.6%), osteoarthritis (112.1%), and other joint disorders. Our finding can be attributed partly to that inflammatory polyarthropathies, such as rheumatoid arthritis (RA), lupus erythematosus, and osteoarthritis, are generally more prevalent in females [3,32,33].

In the UK, osteoarthritis has a prevalence rate of 10.7% and an incidence rate of 6.8% per 1,000 persons per year [34]. Osteoarthritis incidence and prevalence rates increased in older women in the past two decades, with an annual rate of 1.4% [34]. Our analysis found osteoarthritis as the second most common cause of hospital admissions.

RA is a chronic systemic inflammatory condition associated with increased morbidity. In a subset of patients, long-term remission remains challenging to achieve [35]. Nonetheless, with the advent of novel therapeutics in RA, there has been a reduction in disability attributed to arthritis, as well as improved survival. With the improvement of survival rate in these patients, other comorbid conditions described with RA, such as cardiovascular, hematologic, respiratory, and gastrointestinal diseases, have emerged as significant contributors to its morbidity [3]. Furthermore, the advancement in therapeutic interventions used in RA, including biological therapies, has potentially contributed to increased hospital admissions due to associated adverse events. RA therapies render patients immunocompromised and at increased risk of serious infections [36]. In addition, patients with RA are more likely to suffer from depression, anxiety, social impairment, and disability [35]. The abovementioned conditions can increase hospital admissions in RA and other connective tissue diseases.

Another notable increase in hospital admissions was seen among bone density and structure disorders, which was 132.2% between 1999 and 2019. Osteoporosis is highly prevalent in postmenopausal women. The incidence of subsequent fragility fractures also increases with aging [37]. Moreover, there is poor compliance with osteoporosis therapy in the UK [38]. Such poor compliance can increase the risk of fragility fractures. In 2019-2020, there were 60,575 hip fractures in people aged 65 years and above in England [38].

Our study found that males experienced more frequent hospital admissions for infectious arthropathies, disorders of muscles, other dorsopathies, and other disorders of the musculoskeletal and connective tissue than females. The admission rate for infectious arthropathies increased by 114.5%. The common causes of septic arthritis are penetrating trauma and injection drug use [39]. Preexisting joint disease such as RA is another significant risk factor [39]. The UK continues to suffer from intravenous drug use addiction, with an estimated 30,000 intravenous drug users needing health care evaluation for injection site infections, with 18,500 of them requiring hospitalization, as estimated in 2008 [40].

Our study showed that admission rates secondary to ICD-10 categories intraoperative and post-procedural complications increased suddenly in 2015. Before 2015, intraoperative and post-procedural complications were coded under ICD category complications of surgical and medical care, not elsewhere classified (996-999) in ICD-9. This was changed in 2015, explaining the sudden increase in hospitalization rates from this category [41].

Lastly, our study highlights the significant burden of musculoskeletal and connective tissue diseases on the inpatient sector in England and Wales. In the UK, musculoskeletal and connective tissue diseases accounted for 22% of the total burden of illness [42]. It was one of the most frequently recorded diagnoses for hospital admissions in England in 2017-2018, accounting for 7.9% of all admission [42]. The healthcare expenditure for musculoskeletal diseases was estimated to be 4.76 billion euros annually by NHS in 2011 [43]. Close and regular monitoring by primary care physicians, rheumatologists, and orthopedics is necessary for patients with musculoskeletal and connective tissue diseases to detect early disease flares and therapy-related adverse events. Rapid intervention in early flares of diseases and optimized pain control can decrease the need for emergent hospitalization. Patient-centered approaches, including lifestyle changes, smoking cessation, weight loss for obese patients, and physical activity, are pivotal [44]. These can decrease the progression of musculoskeletal disorders, minimize physical disability, and potentially reduce hospital admissions.

Our study has several limitations. It is an ecologic study; hence, patient-level information is unavailable, and our estimates are grouped [45]. Moreover, we do not have information on many variables, which can lead to confounding [45]. Variation in recording hospitalization frequency and their causes might result in bias. During the first 10 years of study, the musculoskeletal system and connective tissue disease hospitalization rate was higher than the last 10 years. An improvement in recording disease frequency with time could explain this increase in the hospitalization rate. Other important limitations are the lack of specificity of ICD-10 codes (i.e., data are presented in the form of a four-character code that lacks specificity) and the inability to determine the exact cause of increased hospitalization for conditions. Moreover, misclassification due to inaccurate or variable coding across different sites can lead to bias. Changes in disease management might shift the treatment of certain disorders from inpatient to outpatient settings and vice versa, and this can also introduce inaccuracy. Because of the lack of other studies providing a comprehensive analysis of all ICD-10 codes related to admissions, we could not compare our methodology to other empirical assessments. Changes in ICD-10 coding is another limitation. The use of ICD-10 in the UK was mandated in 1995, and since then, it has been updated on several occasions [45]. The most recent update was the fifth version and was implemented on April 1, 2016 [45]. Another limitation is the wide range of the age groups in each category provided by the databases. Due to the nature of the data (on a population level) provided by the two databases, we were unable to assess the risk factors that may impact hospital admission nor were we able to adjust our estimates by age groups and gender to account for between-group exposure heterogeneity. Because of the above, our study findings should be interpreted with understanding of its limitations.

## Conclusions

Over the last two decades, the rate of hospitalization for musculoskeletal and connective tissue diseases has increased rapidly and then stabilized or declined in many of the subcategories in England and Wales. Due to the chronicity of these conditions and consequential long-term disability, understanding these trends is essential for national planning to mitigate the associated cost of hospitalization. Further studies are warranted to identify hospitalization risk factors for musculoskeletal and connective tissue diseases.
